# ASSESSMENT OF THE LEVEL OF SATISFACTION OF PATIENTS SUBMITTED TO LATARJET SURGERY UNDER OUTFIT SYSTEM COMPARED TO HOSPITAL SYSTEM

**DOI:** 10.1590/1413-785220233105e264837

**Published:** 2023-10-23

**Authors:** LEANDRO MASINI RIBEIRO, FILLIPE AGRA DE OLIVEIRA COSME, PAULO HENRIQUE SCHMIDT LARA, ALBERTO DE CASTRO POCHINI, BENNO EJNISMAN, PAULO SANTORO BELANGERO

**Affiliations:** 1 Universidade Federal de São Paulo, Escola Paulista de Medicina, Centro de Traumatologia do Esporte, São Paulo, SP, Brazil.; 2 Universidade Federal de São Paulo, Escola Paulista de Medicina, Departamento de Ortopedia e Traumatologia, São Paulo, SP, Brazil.

**Keywords:** Orthopedic Surgery, Ambulatory Surgery, Patient Satisfaction, Joint Instability, Glenohumeral Subluxation, COVID-19, Cirurgia Ortopédica, Cirurgia Ambulatorial, Satisfação do Paciente, Instabilidade Articular, Luxação Glenoumeral, COVID-19

## Abstract

Anterior shoulder instability causes functional changes that affect patients’ quality of life. The Latarjet procedure is one of the most frequently performed surgeries for cases of recurrent shoulder instability.

**Objective:**

To assess the level of satisfaction of patients who underwent the Latarjet procedure in outpatient settings (day hospital) compared with inpatient settings.

**Methods:**

A questionnaire was administered to both groups and a descriptive analysis of the results was performed.

**Results:**

51 patients were included, with a mean age of 29.9 years, 82.3% men and 17.6% women. Of the patients who underwent surgery in the day hospital, 46.1% were operated within 100 days of their first outpatient visit; among those in the inpatient group, 76.3% underwent surgery more than 200 days later. Delays occurred in 15.3% of cases in the day hospital compared with 68.4% in the inpatient group. Of the patients in the day hospital, 92.3% felt comfortable contacting the medical team in case of complications and would perform the procedure again in the same setting. Moreover, 63.2% of inpatients would have preferred to have been discharged on the same day. The final satisfaction rate for both groups was 100%.

**Conclusion:**

Outpatient surgery guarantees more patient comfort, safety, and can be performed in a timely manner and with fewer delays, which has influenced patients’ decision to have surgery during the COVID-19 pandemic. ***Level of Evidence V, Cross-sectional Study.***

## RESUMO

A instabilidade anterior do ombro acarreta alterações funcionais que impactam a qualidade de vida do paciente. A cirurgia de Latarjet é um dos procedimentos mais executados para casos de instabilidade recorrente de ombro.

**Objetivo:**

Comparar o grau de satisfação dos pacientes submetidos ao procedimento de Latarjet no regime ambulatorial (hospital dia) com o dos operados no regime hospitalar.

**Métodos:**

Um questionário foi aplicado em ambos os grupos e uma análise descritiva dos resultados foi realizada.

**Resultados:**

Foram incluídos 51 pacientes, com idade média de 29,9 anos, sendo 82,3% homens e 17,6% mulheres. Dos submetidos à cirurgia no hospital dia, 46,1% operaram em até 100 dias depois do primeiro atendimento ambulatorial; já entre os do grupo hospitalar, 76,3% operaram mais de 200 dias depois. O atraso na cirurgia ocorreu com 15,3% dos pacientes do hospital dia contra 68,4% do grupo hospitalar. Do hospital dia, 92,3% pacientes sentiram-se confortáveis em contatar a equipe médica em caso de intercorrências e fariam novamente o procedimento de forma ambulatorial. Além disso, 63,2% dos internados gostariam de ter recebido alta no mesmo dia. O grau de satisfação final em ambos os grupos foi de 100%.

**Conclusão:**

A cirurgia ambulatorial garante mais conforto para o paciente, mostrando-se segura e podendo ser performada em tempo hábil e com menos atrasos, o que influenciou a decisão dos pacientes em operar durante a pandemia de COVID-19. **Nível de Evidência V, Estudo Transversal.**

## INTRODUCTION

Anterior shoulder instability is a spectrum of anatomical and functional changes that lead patients to experience recurrent loss of joint congruence or apprehension when performing activities, reducing their ability to perform basic daily functions and consequently their quality of life. The Latarjet procedure is one of the most performed surgeries for recurrent shoulder instability, consisting of transferring the coracoid to the anterior glenoid, preventing new episodes of dislocation and increasing patient safety when performing tasks.^1^

More than 50 years ago, this type of procedure could only be performed in a hospital, thus, patients had to be hospitalized and discharged after a few days. They often had to wait for months until the day of surgery due to the limited number of beds or the demand for other emergencies that needed to be dealt with sooner.^2^

The concern of doctors and managers about the problems faced in performing surgeries is not new, especially in the current scenario, in which most hospital beds are allocated to COVID-19 patients, increasing the waiting list for orthopedic procedures. Studies from the beginning of the last century show patients who underwent surgery and returned home the same day, giving rise to the concept of outpatient surgery (also known as day hospital).^3^ In the 1970s, the number of facilities and associations focused on the study of outpatient surgery increased, seeking to improve efficiency, convenience in care, and consequently, patient satisfaction.^4^

This study aimed to assess the level of satisfaction of patients who underwent the Latarjet procedure in outpatient settings compared with inpatient settings.

## METHODS

This descriptive, observational, and cross-sectional study applied an informed consent form and a structured questionnaire on the level of satisfaction of patients who underwent outpatient surgery, based on a review article,^5^ combining statements about the preoperative period, the day of surgery, and the postoperative period. The questionnaire was also adapted for patients who underwent inpatient surgery.

The patients assessed were divided into two groups: the first underwent outpatient surgery and the second inpatient surgery. Both groups were operated by the same team of surgeons.

**Figure 1:**
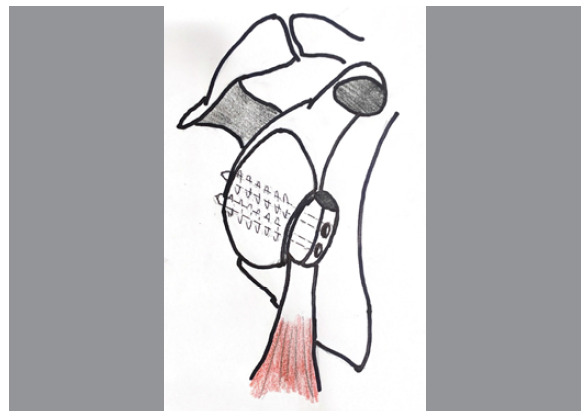
Latarjet technique for fixing the coracoid process in the anteroinferior glenoid.

The surgery performed was the open Latarjet procedure for anterior glenohumeral instability, which consists of transferring and fixing the coracoid process to the anteroinferior glenoid, acting with a triple biomechanical effect: anterior bone block, the sling effect caused by the joint tendon, and the stability caused by the repair of the coracoacromial ligament in the capsule^6^ (Figure 1). All patients underwent a regional brachial plexus block as anesthesia for the surgery.

All patients were discharged from the hospital with the same analgesic guidelines and prescriptions for anti-inflammatories, simple analgesics, and opioid analgesics.

Before the questionnaire was administered, the project was sent to the Research Ethics Committee, which approved it under number CAAE 89698818.5.0000.5505.

The platform used for data collection was Google Forms. The answers were stored in a database and analyzed using SPSS software. The values are expressed as mean ± standard deviation. Pearson’s Chi-squared test was used to compare the frequency of the variables. The percentage of postoperative pain in both groups and age were analyzed using one-way analysis of variance (ANOVA). The impact of patient satisfaction was assessed using linear analysis.

## RESULTS

This study collected data from 51 individuals. The relevant independent variables included in the analysis were sex, age, waiting time until surgery, comfort with having surgery during the COVID-19 pandemic, and information, guidance, and attitude of the team in general before, during, and after the procedure.

The mean age was 29.9 ± 7.1 years, 42 men (12 in the outpatient group and 30 in the inpatient group) and nine women (only one in the outpatient group and eight in the inpatient group).

We found no significant differences in pain assessment between the two groups. The expected pain in the first postoperative week, assessed using the visual analogue scale (VAS), was 6.45 ± 1.79.

Regarding the time until surgery in relation to the first outpatient visit, four patients waited < 50 days (day hospital), three waited 50 to 100 days (two in the outpatient group and one in the inpatient group), 11 waited 100 to 200 days (only three in the outpatient group and eight in the inpatient group), 31 waited > 200 days (two in the outpatient group and 29 in the inpatient group), and only two patients (outpatient group) did not remember. In total, 76.3% of patients who underwent inpatient surgery waited > 200 days and 21.1% waited 100 to 200 days. Moreover, 46.1% of patients in the outpatient group waited < 100 days (Table 1).

**Table 1:** Estimated waiting time until the day of surgery for patients who underwent outpatient (day hospital) and inpatient surgery.

Estimated waiting time	Inpatient surgery	Day hospital
< 50 days	0	4
50-100 days	1	2
100-200 days	8	3
> 200 days	29	2
Don't remember	0	2

For 53.8% of outpatients, the procedure performed in the day hospital had a positive influence on their decision to have surgery during the COVID-19 pandemic. However, 76.9% of all patients were indifferent to the decision to have surgery during the pandemic.

Only 15.7% of patients became more anxious after receiving information about the surgery, and 92.1% felt that they had fully understood the information. In total, 54.9% believed that the best time to receive this information was a few days before surgery, while for 21.5%, the best time was a few weeks before. Eight patients were indifferent as to the best time.

**Figure 2:**
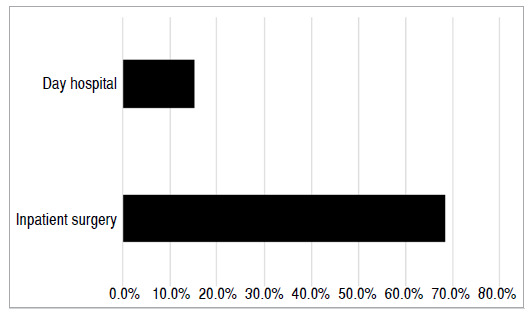
Average delay in surgery among patients who underwent outpatient (day hospital) and inpatient surgery.

There was a significant difference and a greater chance of delays in inpatients than in outpatients (p < 0.01). In total, 15.3% of patients reported a delay in outpatient surgery, but considered the waiting time to be reasonable. In inpatient surgery, 68.4% reported a delay (Figure 2) and considered the time to be long but acceptable.

In this study, 90% of the patients fully trusted the health professionals responsible for their treatment and 98% had prior contact with the nurses or doctors who would be in the operating room before undergoing surgery. For 64.7%, this contact completely relieved their anxiety, while for 21.5%, the contact partially relieved their anxiety and 11.7% were indifferent.

Regarding the attitude of the medical and nursing teams and other professionals, no significant differences were found between the groups. Almost all patients rated the professionals 4 and 5. The characteristics evaluated by the patients were friendliness, availability, sensitivity, concern, professionalism, and attention.

Care after discharge was the same or simpler than expected for 100% of outpatients and 92.1% of inpatients. Only three patients complained that care was more difficult than expected.

Regarding contact with the medical team in case of complications, there was no significant difference (p < 0.01). Of the patients who underwent outpatient surgery, 92.3% felt comfortable, while in the inpatient group, the opposite was true: 94.7% did not feel comfortable using the contacts provided.

Regarding postoperative complications, only three patients in the inpatient group had to return to the unit after discharge, two due to pain and one due to superficial infection of the surgical wound (Table 2). The complaints were resolved in the emergency room, without the need for further hospitalization. For 89.4%, the medications administered in the postoperative period were sufficient for pain relief.

**Table 2:** Postoperative complications and the need to return to the unit.

	Inpatient surgery	Day hospital
Pain	5.2%	0%
Superficial infection	2.6%	0%
No complications	92.1%	100%

In the outpatient group, 92.3% would undergo the procedure again in the proposed setting and only one reported he would prefer to be hospitalized. Of the inpatients, 63.2% would have preferred to undergo outpatient surgery, without the need for hospitalization. In both groups, the final level of satisfaction with the surgery in general was 100%.

## DISCUSSION

Regarding the epidemiology of shoulder instability, the age of the patients corresponds to the age range in the literature:^7^ generally young, active adults, who are also men in most cases of recurrent dislocation.^8^

The waiting time from the first outpatient visit to the day of surgery was shorter in the day hospital group. In almost half of the cases, the interval between the first visit and surgery was three months. This process took almost twice as long in more than half of the patients who underwent inpatient surgery. According to the literature, the average waiting time for outpatient surgery is 100 days and for inpatient surgery 140 days,^9^ which is similar to the findings of this study.

The COVID-19 pandemic was also a factor that led to a large reduction in surgeries, due to both the reduction in beds for surgical patients and patients’ fear of coming into contact with hospital services and running the risk of infection.^10^ The absence of the need for hospitalization led almost half of the patients who underwent outpatient surgery to accept the waiting time and be more relaxed about having the procedure during the pandemic.

Another relevant point in patient care is the provision of information about the procedure. Almost all patients were able to understand the pathology, the risks and benefits of surgery, and how it would be performed, and for more than half, this information should be provided a few days before surgery. Therefore, going for a consultation a few days before surgery can further reassure patients. Another factor that proved to reduce patients’ anxiety was the prior conversation before entering the operating room. For more than half of patients, this was a protective factor for anxiety.

As day hospital services operate during business hours, the number of procedures performed is limited and they need to be scheduled and organized on the surgical map, leading to punctuality in almost all cases. In inpatient settings, despite the existence of a team that coordinates the surgical schedule, unforeseen events, such as emergency surgeries, and delays in scheduled procedures can always occur, as the results show. However, the medical and nursing teams are on an equal footing in both services, scoring highly in the subjective criteria, which shows that the care provided to patients is practically the same.

Patients’ pain was the same in both groups in the postoperative period, which was expected to be higher for the patients operated in the day hospital, either because they believed they would not have stricter control of their medication schedules or because they felt postoperative instructions was more difficult. This was not true, since for most patients the care would be simpler or identical to what they expected.

Also, regarding hospital discharge, a relevant piece of information was the comfort of using emergency contacts. Patients who underwent inpatient surgery did not feel comfortable calling or texting a member of the medical team, which can be explained by the presence of a backup team (nurses, technicians, and assistants) providing care. The opposite was true for patients who underwent outpatient surgery, as almost all of them felt comfortable contacting the medical team, if necessary, since they did not have emergency support in case of complications.

Postoperative complications were minimal, which is expected for the procedure. The main complaint was pain, which in almost all patients was well controlled with the prescribed analgesia.

Finally, almost all outpatients would undergo the procedure again in the same setting and recommend it to friends or family. On the other hand, in the inpatient group, more than half would have preferred to be discharged on the same day, which reinforces the safety of the Latarjet procedure even in the immediate postoperative period.

## CONCLUSION

Although satisfaction with the outcome of the Latarjet procedure was very good in both groups, the experience of patients who underwent outpatient surgery was better for the patient, with fewer delays, timely performance, and less preoperative anxiety, especially during the COVID-19 pandemic.
